# Radiation Can Regulate the Expression of miRNAs Associated with Osteogenesis and Oxidation in Exosomes from Peripheral Blood Plasma

**DOI:** 10.1155/2021/6646323

**Published:** 2021-02-15

**Authors:** Yu Du, Haiyang Tang, Xia Gu, Yixin Shi, Ping Gong, Yang Yao

**Affiliations:** ^1^State Key Laboratory of Oral Diseases, National Clinical Research Center for Oral Diseases, Department of Oral Implantology, West China Hospital of Stomatology, Sichuan University, Chengdu, China; ^2^Chengdu Second People's Hospital, Department of Stomatology, China; ^3^Stomatological Hospital of Chongqing Medical University, China

## Abstract

**Objectives:**

Radiotherapy is a common therapy in head and neck tumors, which may cause a side effect radiation bone injury (RBI). Furthermore, it has been investigated that microRNA (miRNA) expression levels were altered after radiotherapy. Exosomes play a role in bone formation as miRNA containers, while radiation affects exosomes composition, secretion, and function. So, our objective is to explore changes in miRNA levels during bone formation after radiotherapy and identify the differentially expressed miRNAs (DE-miRs) in plasma exosomes during the process of osteogenesis related to irradiation.

**Materials and Methods:**

In this study, we analyzed nine samples from three rabbits exposed twice to radiation (15 Gy each) and detected DE-miRs from irradiated plasma exosomes during the process of osteogenesis by RNA sequencing. Further, we identified DE-miRs with significant differences and predicted their target genes via the bioinformatics analysis tools Targetscan v7.2 and miRPathDB v2.0. Finally, we identified radiation-responsive miRNAs and predicted their target genes during osteogenesis.

**Results:**

Taken together, we have identified some DE-miRs in irradiated plasma exosomes, which were involved in several vital signaling pathways related to bone physiology, such as the Wnt pathway, MAPK cascade, and calcium modulating pathway.

**Conclusions:**

We have found that plasma exosomes are one of the ways by which radiation can affect bone metabolism and regeneration. However, the specific mechanisms of how these plasma exosomal miRNAs mediate the osteogenesis pathways must be further investigated. *Clinical Relevance*. Radiotherapy may cause radiation bone injury, and miRNA expression levels in rabbit plasma exosomes are altered after radiotherapy. High-throughput RNA sequencing can identify the differentially expressed miRNAs in irradiated plasma exosomes during the process of osteogenesis. These findings make sense to develop novel therapeutic strategies for treating radiation-induced bone injury disorders.

## 1. Introduction

Radiotherapy is the most frequently used method for nonsurgical treatment of head and neck tumors, the sixth most common systemic tumors [1]. However, radiotherapy can cause radiation bone injury (RBI) [2] as well, which can result in radiation-induced osteomyelitis, osteoradionecrosis, and even pathologic fracture, from which patients would suffer a poor quality of life. With regard to RBI, there are no currently available preventive or treatment measures for use clinically. Therefore, it is essential to investigate irradiation-induced changes to the bone under current treatment regimens.

In recent years, studies showed that miRNA (microRNA) expression levels were altered after radiotherapy of head and neck squamous cell carcinomas and other tumors, suggesting that radiation may adversely affect miRNA levels [3–6]. These affected miRNAs compose a class of noncoding, endogenous, small, single-stranded RNAs found in eukaryotes, which negatively regulate target mRNAs posttranscriptionally by shearing or inhibiting their translation [7]. For instance, miR-200b and miR-155 were demonstrated to serve as predictive biomarkers of the efficacy of chemoradiation in locally advanced head and neck squamous cell carcinomas [8]. Furthermore, other studies have shown that stem cell osteogenesis is strictly regulated by miRNAs, including the main signaling pathways [9, 10] and related transcription factors [11, 12].

Exosomes can enter target cells by pinocytosis as secreted microvesicles [13], which allows the exchange of miRNAs and other genetic information directly between cells and plays a unique role in long-distance cellular communication [14].

Exosomes have also been shown to stimulate stem cell differentiation [15] and promote the repair of bone defects in both healthy [16] and osteoporotic animals [17].

Moreover, exosomes secreted from irradiated cells are assumed to function through bystander effect, which means exosomal miRNA can be transported between irradiated cells and nonirradiated cells [18, 19]. One study reported that not only the composition and secretion of exosomes released from donor cells were affected by radiation, but the functions of recipient cells receiving exosomes from irradiated cells were affected as well [20]. Some representative soluble signaling factors were found including reactive oxygen species (ROS) and nitric oxide, as well as secondary messengers such as calcium fluxes and cytokines. However, the mechanism by which radiation affects exosome composition, secretion, and function is poorly understood [21]. Because of the convenience of collection and amplification capacity of plasma compared to those of other cell sources, the use of plasma exosomes has attracted much attention [22, 23]. However, there are few studies concentrated on how irradiation affects miRNA levels in plasma exosomes. With the help of RNA sequencing, we were able to distinguish changes in miRNA expression and identify differentially expressed miRNAs (DE-miRs). In addition, exploring the target genes of these DE-miRs allowed us to investigate significant changes in bone metabolism caused by irradiation.

This study is aimed at identifying the DE-miRs in plasma exosomes during the process of osteogenesis related to irradiation and explore changes in miRNA levels during bone formation after radiotherapy. The goal of the study was to develop improved treatment regimens for patients suffering from RBI-induced symptoms after radiotherapy.

## 2. Materials and Methods

### 2.1. Materials and Experimental Design

We obtained eight healthy male adult New Zealand white rabbits numbered from 1 to 8, each weighing three to four kilograms. These rabbits were from the Huaxi Animal Center of Sichuan University. Rabbits were fed of proper amount of food every day and maintained at a temperature of 25°C in standard cages, which ensured the same growing environment. All rabbits were observed and recorded every three days for two months. Each rabbit was anesthetized by an intravenous injection of sodium pentobarbital at the dose of 30~35 mg/kg into the ear vein. A surgical drape was placed to expose the abdomen and thighs, and a hole measuring 30 × 10 cm was made in the drape to serve as the irradiation window. The irradiation was administered using a 60Co-*γ*-ray source at the Seventh People's Hospital of Chengdu, China. We fixed each rabbit on an irradiation table to ensure that the irradiated area remained stationary to guarantee a consistent radiation effect. The radiation distance and rate were 110 cm and 0.83 Gy/min, respectively. Rabbits received an initial dose of 15 Gy (equivalent to a single dose of human radiation) and a second dose of 15 Gy after one month, racking up to the cumulating dose of 30 Gy. After that, rabbits were intramuscularly injected with penicillin at the dose of 80,000 IU/kg twice a day for three days.

### 2.2. Extraction of Plasma Exosomes

We intravenously anesthetized each rabbit on three separate occasions: before every radiotherapy, seven days after receiving the first dose of radiation to collect plasma, and seven days after receiving the second dose to collect plasma. Each rabbit received 4 times of anesthesia totally. We placed each rabbit in the supine position and fixed the limbs on an animal operating table after anesthesia. Then, we shaved and disinfected the chest area and extracted 14–16 milliliters of blood from the heart. It was essential for us to make sure that all operations except for intervening factors were uniform. After all experiments were finished, rabbits were humanely euthanized using intravenous injection of sodium pentobarbital at the dose of 100 mg/kg. The experiment involved ethical part conforms to the scientific experiment ethical requirements, which is agreed by Research Ethics Committee of West China Hospital of Stomatology. Besides, its reference number is WCHSIRB-D-2017-050.

The rabbit plasma was mixed with thrombin, incubated for 5 min at room temperature, and centrifuged at 10,000 × g for 5 min to remove fibrin and 3000 × g for 15 min to discard most platelets and cell debris. And after centrifuging the plasma, we used the ExoQuick™ Exosome Precipitation Solution (Palo Alto, CA, USA) to obtain plasma exosomes and preserved them at -80°C. We randomly selected three exosome samples (numbers 4, 7, and 8) to undergo miRNA gene chip high-throughput sequencing for a comparison of miRNA levels before and after radiotherapy. For example, for rabbit #4, the plasma exosome samples were coded as A4 (collected from plasma before radiotherapy), B4 (collected from plasma after the first dose), and C4 (collected from plasma after the second dose).

### 2.3. Small RNA Library Construction and High-Throughput Sequencing

**Figure 1:**
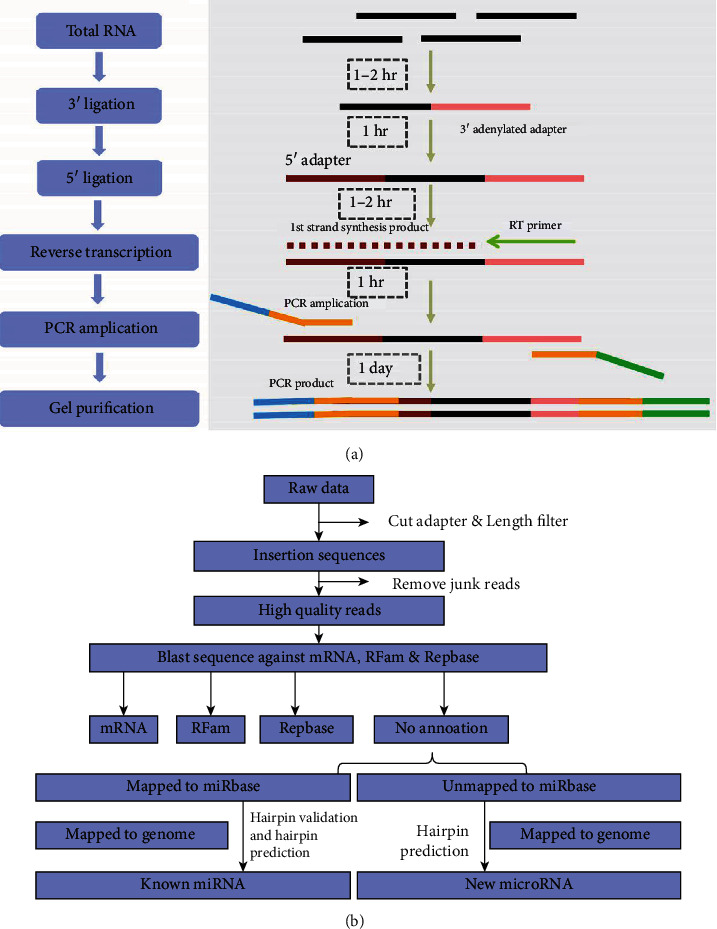
Schematic of sequencing process of RNAs. (a) Construction of the small RNA library. (b) Small RNA data analysis process.

As shown in Figure 1(a), the small RNA sequencing library was prepared using the TruSeq Small RNA Sample Prep Kit (Illumina, San Diego, USA). Then, we employed the Illumina Hiseq2500 to sequence the constructed libraries by single-end sequencing (50 bp) at the LC-BIO (Hangzhou, China) following the recommended protocol, and 3 repetitions were made in the sequencing.

### 2.4. Identification of miRNAs

As illustrated in Figure 1(b), we compared the remaining sequences to various RNA sequence databases (excluding miRNAs), such as mRNA and RFam (containing rRNAs, tRNAs, snRNAs, snoRNAs, etc.), and the repeat sequence database (Repbase). Specially, miRNAs were identified as new 5p or 3p miRNA candidate sequences if the detected sequence was comparable to the corresponding arm site of a known miRNA. However, those unmatched sequences were compared to precursors of other species in miRBase21.0 using the Bowtie aligner a second time. The matched miRNA precursor sequences were compared to the genomic sequences of the sequenced species to confirm the identities of reported miRNAs.

### 2.5. miRNA Target Prediction and Function Analysis

Target genes of the DE-miRs in exosomes were predicted using the Targetscan online tool from which we also obtained gene annotation data. The efficiency of the miRNA target sites predicted by this software was scored and ranked by the aggregate *P*_CT_ method (the probability of conserved targeting, as described by Friedman et al. [24], has been calculated for all highly conserved miRNA families).

As each DE-miR targeted multiple genes, only genes with an aggregate *P*_CT_ score less than 1.2 were considered targets. In addition, default parameters for target prediction were used. We used another online tool miRPathDB v2.0 to infer the association between the target gene and the miRNA signaling pathway. Here, we chose two-tailed *t*-test to analyze the miRNA differential expression based on normalized deep-sequencing counts. The significance threshold was set to be 0.01 and 0.05 in each test.

## 3. Results

### 3.1. Overview of Small RNAs from Exosomes via High-Throughput Sequencing

We obtained unique sequences (uniq) from the sequencing data and the corresponding number of copies of each unique sequence. After comparisons, we classified different kinds of sequences into various categories. Thereinto, the valid reads were regarded as undisclosed and further used for miRNA identification and prediction analysis.

Taking sample A4 as an example, we constructed the Table 1 shown below. Besides, we compared the total sequencing data (total) and unique data (number of kinds) of each sample with the RNA family (RFam, including rRNA, tRNA, snRNA, and snoRNA etc.) database and the Repbase database, respectively, for further identification of miRNAs.

### 3.2. Valid Data Analysis and Length Distribution

After data processing, we gathered the valid data from each sample in Table 2.

**Figure 2:**
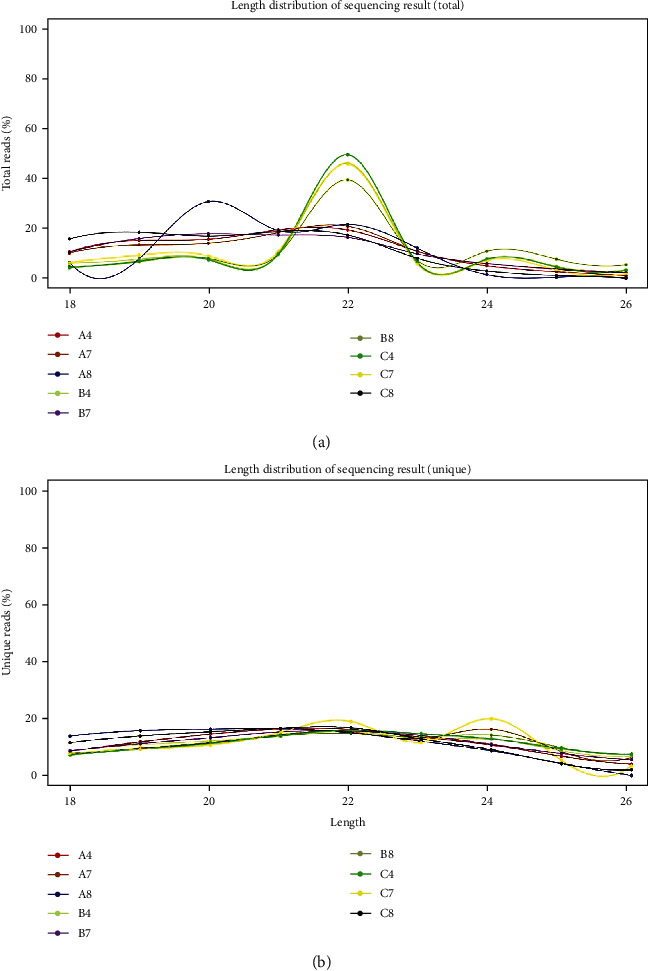
Length distribution of sequencing results (total; unique). Legends: A4, A7, A8, B4, B7, B8, C4, C7, and C8.

We further calculated the length distribution of all valid data and the number of unique sequences. As shown in Figure 2, most of the data were distributed between 20–24 nt, which is typically observed after Dicer enzyme cleavage.

### 3.3. The Length Distribution of Detected miRNAs

Compared with the miRNA database (miRBase 21, released in June 2014), we detected 421 kinds of miRNAs, 25 of which were novel miRNAs. Based on a statistical analysis of the detected miRNAs, we obtained the number of unique miRNA sequences of different lengths and their distribution in Table 3, with most miRNAs concentrated in the range of 20–24 nt, which was consistent with the definition of miRNA. However, according to the species specificity of rabbits, we did not pay specific attention to these novel miRNAs. We focused on miRNAs related to oxidative stress and osteogenic pathways rather.

### 3.4. Identification of Differentially Expressed miRNAs

**Figure 3:**
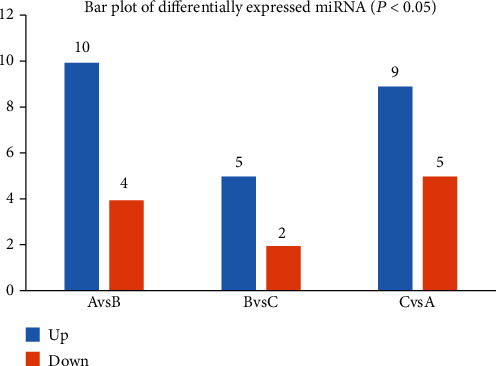
A bar plot of the DE-miRs in different comparison groups. Legends: up and down.

We applied a *t*-test to assess significantly different miRNAs and set the screening threshold (*P* value <0.05) for the differential expression analysis of the detected miRNAs. Under the inclusion criteria mentioned above, a total of 29 DE-miRs were identified. Additionally, there were 14, 7, and 14 DE-miRs in A vs. B, B vs. C, and A vs. C, respectively, as described in Figure 3.

**Figure 4:**
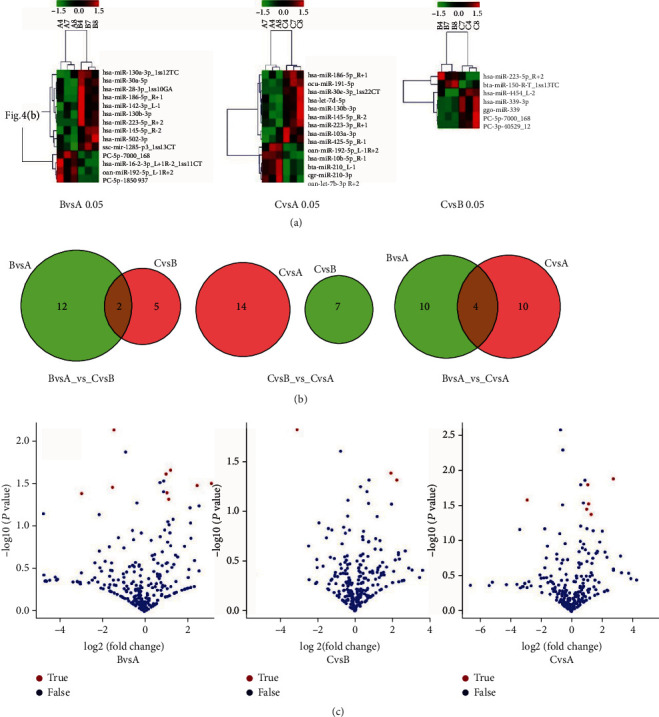
(a) DE-miRs in different comparison groups. (b) Identical and unique DE-miRs between the different comparison groups. This can visually display the results. For BvsA_vs._CvsB, there were 2 identical miRNAs, for CvsB_vs._CvsA, there was no identical miRNA existing, and for BvsA_vs._CvsA, there were 4 identical miRNAs detected. (c) The statistical significance of the changes in miRNA expression. The abscissa represented the variance in miRNA expression in the different experimental groups, while the ordinate represented the statistical significance of the changes in miRNA expression. The blue dots were miRNAs with no significant differences, and the red dots were miRNAs with significant differences. According to the results, we could acquire the information that, regarding the expressions of miRNAs with significant differences in different comparisons, 3 were downloaded while 6 were uploaded in comparison of BvsA, 1 was downloaded while 2 were uploaded in comparison of CvsB, and 1 was downloaded while 6 were uploaded in comparison of CvsA.

To assess these DE-miRs, we utilized differential gene cluster analysis to observe their up- and downregulation as shown in Figure 4(a). In this analysis, we used the spacing of 3 kb, 5 kb, 10 kb, or 50 kb to perform cluster analysis, following further exploration of clusters whose number of miRNAs within a distance range greater than 2.

In addition, miRNAs with similar expression patterns located closer in the same atlas. Red stripes indicated high expression; green stripes indicated low expression. As illustrated in Figure 4(b), these experiments allowed us to identify significantly differences in expressed genes between the samples, which were then used to construct Venn diagrams to further reveal identical and unique DE-miRs between the different comparison groups. Moreover, taking the fold change in expression into consideration, which represents the expression level, we set the criteria for log_2_ fold change to be greater than 1 and the *P* value to be less than 0.05. In reality, 15 DE-miRs were thus identified. Then, we generated a volcano diagram of DE-miRs in Figure 4(c).

As presented in the diagrams, there were nine red dots in the comparison of A and B samples: PC-5p-7000_168, ssc-mir-1285-p3_1ss13CT, hsa-miR-30a-5p, hsa-miR-186-5p_R+1, hsa-miR-28-3p_1ss10GA, PC-5p-1850_937, hsa-miR-130b-3p, hsa-miR-16-2-3p_L+1R-2_1ss11CT, and hsa-miR-142-3p_L-1. Similarly, there were three red dots in the comparison of B and C samples: hsa-miR-223-5p_R+2, PC-5p-7000_168, and hsa-miR-4454_L-2. Finally, there were six red dots in the comparison of A and C samples: hsa-miR-186-5p_R+1, ocu-miR-191-5p, oan-let-7b-3p_R+2, hsa-miR-145-5p_R-2, hsa-let-7d-5p, and hsa-miR-130b-3p. The prefix “PC” signifies a novel miRNA.

### 3.5. Targets of Differentially Expressed miRNAs and Functional Analyses

The mature sequences of the 15 miRNAs in plasma exosomes, which were affected by irradiation, were utilized to search for their target genes. As for the miRNAs whose targets were unclear, we had the advantage of the online tools Targetscan v7.1 and miRPathDB v1.0 to predict their targets, and the results are shown in Table 4. Additionally, as our research did not concentrate on the discovery of novel miRNAs, the two novel miRNAs were excluded. These predicted targets had a variety of functions, including those involved in cell cycle and proliferation, angiopoiesis, adipogenic differentiation, and bone formation [25–27].

## 4. Discussion

In the present study, 15 key DE-miRs were identified after irradiation, including 10 upregulated miRNAs and 5 downregulated miRNAs. Interestingly, let-7b and let-7d were present in the same miRNA family (let-7) but showed distinct regulation patterns in C samples as compared to those observed in samples of A. Furthermore, there were two miRNAs (miR-186 and miR-130) that maintained similar regulation patterns in response to different radiation doses, which may be of significance during osteogenesis. Several potential target genes for miR-186, miR-130, and let-7 were predicted using online websites for target prediction. Further investigation revealed that these targets were related to osteogenic and chondrogenic differentiation, maintenance of stemness, and adipogenic differentiation.

The let-7 miRNA family is highly conserved among different animal species, which suggests that it is likely to play important and similar roles in the physiological processes of disparate species [28, 29]. Since miRNAs negatively regulate their target genes, it is expected that targets of radiation-induced upregulated miRNAs may cause aberrant physiological processes. For example, let-7 was found to regulate cell proliferation during the evolution of lung cancer, indicating that its downregulation facilitated carcinogenesis [30]. In this study, we obtained two significant let-7 isoforms: let-7b and let-7d. The expression pattern of let-7d was consistent during the bone formation phase, which suggests that it might be a promotor of osteogenesis and bone development by regulating high-mobility group AT-hook 2 (HMGA2) [31].

HMGA2 is a nonhistone chromosomal protein that functions as an architectural transcriptional factor and is highly expressed in poorly differentiated cells. Further, we confirmed that endogenous protein expression levels of HMGA2 were effectively inhibited by let-7 [32, 33]. These findings indicated that HMGA2 suppresses bone formation and promotes adipogenesis. Additional study is needed to identify the mechanism by which the let-7/HMGA2 axis mediates osteogenesis. It is worth noting that HMGA2 can be affected by BMP4, which is related to the early stages of adipocyte differentiation in mesenchymal stem cells (MSCs) [34]. No definite research has connected let-7b and osteogenesis, and we propose that not all isoforms of let-7 are involved in bone physiology, such as let-7b.

It had been found that miR-130b was overexpressed in both osteo-differentiated MSCs and chondro-differentiated MSCs, suggesting that miR-130b plays an important role in the osteogenic and chondrogenic differentiation of MSCs [35]. Another study revealed that miR-130b targeted naked cuticle homolog 2 (NKD2) and regulated the Wnt signaling pathway, promoting proliferation and inhibiting apoptosis of osteosarcoma cells [36]. NKD2 is well recognized as a negative regulator of the Wnt signaling pathway. The Wnt/*β*-catenin pathway plays a vital role in bone physiology, specifically in bone mass acquisition, differentiation, remodeling, and maintenance [37]. In our research, miR-130b was overexpressed after radiation, which is consistent with prior findings. In addition, the miR-301b/miR-130b/PPAR*γ* axis was also shown to underlie the adipogenic capacity of MSCs derived from different tissues [38].

Researchers have found altered levels of miR-186 in a variety of cancer tissues, which highlight the role of miR-186 in molecular mechanisms of carcinogenesis [39–41]. Furthermore, miR-186 was reported to suppress cell proliferation and inhibit organ metastasis of diverse cancers, including gastric cancer, prostate cancer, and non-small-cell lung cancer [40, 42, 43]. For example, miR-186 suppressed cell proliferation by negatively targeting oncogene GOLPH3 in prostate cancer tissues [40]. Additionally, we predicted miR-186 target genes MAP3K2 and MAPKAPK5 using online tools. A previous study showed that miR-186 suppressed cell proliferation and metastasis by targeting MAP3K2 in a case of non-small-cell lung cancer [42]. The mitogen-activated protein kinase (MAPK) pathway has been shown to regulate proliferation, migration, differentiation, and apoptosis as important cellular processes. Although the MAPK pathway is acknowledged to play a significant part in bone formation, its physiological effects during osteogenesis are unclear. Some research supports its positive role in osteoblast differentiation, while other studies show that this pathway suppresses this process [44, 45]. Furthermore, previous research also showed that p38 MAPK coordinated osteoblastic differentiation via Osx [37]. We speculated that miR-186 may regulate osteoblastic differentiation via the MAPK signaling pathway.

Radiation injury is always associated with cell death, especially apoptosis, which plays a vital role not only in the fields of growth, development, and senescence of human beings, but also in many physiological and pathological processes [46]. Meanwhile, the oxidative stress associated with ROS is demonstrated to be one key link in apoptosis [47]. As mentioned above, the MAPK pathway has been confirmed to be an important pathway in regulating apoptosis. ROS overproduction and/or the antioxidant mechanism reduction, working as the mediating factor, can activate the pathway to control the process of apoptosis. In this research, the change of hsa-miR-186-5p, hsa-miR-142-3p, hsa-miR-223-5p, and hsa-let-7d-5p could explain this point. However, it has been remained unclear yet about the actual molecular mechanisms of apoptosis mediated by ROS. Our research suggested that there would be potential relationships between exosomes from irradiated plasma, which can be regarded to have negative effects on osteogenesis through some pathways concerning ROS. However, further studies are needed to reveal the correlative specific roles of exosomes and ROS in the field of radiation injury and radiotherapy.

## 5. Conclusions

In summary, we have identified miRNAs in plasma exosomes which are altered as response and consequences after irradiation. These miRNAs are suggested to play a regulatory role in cell proliferation, differentiation, and apoptosis, which appear to be typical physiological reactions to a detrimental external environment. Moreover, we suggested that some of these miRNAs related with several vital signaling pathways of osteogenesis, such as the Wnt pathway, MAPK cascade, and calcium modulating pathway, are one of the ways in which radiation could affect biological activities such as bone metabolism. However, the specific mechanisms of how these exosomal miRNAs derived from plasma mediate the osteogenesis pathways need further investigation. These findings make sense to developing novel therapeutic strategies for treating radiation-induced bone injury disorders.

## Figures and Tables

**Table 1:** Sequence distribution of sample A4.

lib	Type	Total	% of total	Uniq	% of uniq
Raw reads	NA	11630099	100.00	512205	100.00
3ADT&length filter	Sequence type	8699950	74.81	348881	68.11
Junk reads	Sequence type	3890	0.03	794	0.16
RFam	RNA class	11747	0.10	527	0.10
mRNA	RNA class	33188	0.29	209	0.04
Repeats	RNA class	383	0.00	71	0.01
Valid reads	Sequence type	2,881,314	24.77	161,773	31.58
rRNA	RNA class	4280	0.04	143	0.00
tRNA	RNA class	855	0.01	104	0.00
snoRNA	RNA class	2174	0.02	19	0.00
snRNA	RNA class	3723	0.03	213	0.00
Other RFam RNA	RNA class	715	0.01	48	0.00

**Table 2:** The distribution of valid data from each sample.

	Total	% of total	Unique	% of unique
A4	2,881,314	24.77	161,773	31.58
A7	2,122,877	22.07	167,930	30.45
A8	5,573,321	57.97	105,031	22.29
B4	4,228,472	40.97	186,193	32.47
B7	2,999,612	29.34	170,509	23.80
B8	1,979,161	18.40	114,312	21.12
C4	4,145,851	42.18	174,921	25.84
C7	4,175,157	40.42	267,438	35.49
C8	2,454,765	24.51	141,241	25.79

**Table 3:** The detected miRNA length distribution statistics.

Length	Unique miRNA	%
18	22	5.23
19	13	3.09
20	28	6.65
21	108	25.65
22	187	44.42
23	51	12.11
24	11	2.61
25	1	0.24
All	421	100.00

**Table 4:** Target genes for irradiation-responsive miRNAs and detailed information about their target genes.

miRNA name	Target gene	Location	Biological process
hsa-miR-30a-5p	POLR3E	Chromosome 16: 22,297,375-22,335,103 forward strand	RNA polymerase III activity
hsa-miR-186-5p	MAP3K2	Chromosome 2: 127,298,730-127,388,465 reverse strand	MAPK cascade
hsa-miR-28-3p	TMEM167B	Chromosome 1: 109,089,803-109,096,934 forward strand	Protein secretion, intracellular transport
hsa-miR-130b-3p	AGO2	Chromosome 8: 140,520,156-140,635,619 reverse strand	Wnt signaling pathway, calcium modulating pathway, miRNA metabolic process
hsa-miR-16-2-3p	RORA	Chromosome 15: 60,488,284-61,229,319 reverse strand	Angiogenesis, negative regulation of fat cell differentiation
hsa-miR-142-3p	MAP4K3	Chromosome 2: 39,249,266-39,437,312 reverse strand	MAPK cascade, response to UV
hsa-miR-223-5p	FGF7	Chromosome 15: 49,423,096-49,488,775 forward strand	Mesenchymal cell proliferation, MAPK cascade, positive regulation of cell proliferation
hsa-miR-4454	CRCP	Chromosome 7: 66,114,604-66,154,568 forward strand	Neuropeptide signaling pathway
ocu-miR-191-5p	CASK	Chromosome X: 41,514,934-41,923,463 reverse strand	Positive regulation of calcium ion import
hsa-miR-145-5p	ROCK1	Chromosome 18: 20,946,906-21,111,851 reverse strand	Rho protein signal transduction, I-kappaB kinase/NF-kappaB signaling
hsa-let-7d-5p	HMGA2	Chromosome 1: 36,339,624-36,385,896 reverse strand	Regulation of MAPK cascade
oan-let-7b-3p ssc-mir-1285	The genes from Ornithorhynchus anatinus and Sus scrofa are still unknown.		

Target genes for irradiation-responsive miRNAs and detailed information about their target genes, including gene location and functional annotation were shown, while two novel miRNAs are not included in this table. They were selected as significantly uploaded (*P* < 0.01) miRNAs, especially clustering in osteogenesis.

## Data Availability

The microarray data used to support the findings of this study are included within the article.
